# Predicting Miscarriage Risk Based on Periodontal and Hematological Parameters Using Artificial Neural Network and XGBoost Models

**DOI:** 10.3390/diagnostics16162634

**Published:** 2026-08-19

**Authors:** Mehmet Özsan, İsa Temur, Katibe Tuğçe Temur, Andaç Batur Çolak

**Affiliations:** 1 Department of Physiology, Faculty of Medicine, Niğde Ömer Halisdemir University, 51240 Niğde, Türkiye; 2 Department of Obstetrics and Gynecology, Faculty of Medicine, Niğde Ömer Halisdemir University, 51240 Niğde, Türkiye; isatemur@ohu.edu.tr; 3 Department of Oral and Maxillofacial Radiology, Faculty of Dentistry, Niğde Ömer Halisdemir University, 51240 Niğde, Türkiye; tugcetemur@ohu.edu.tr; 4 Department of Information Systems and Technologies, Faculty of Computer and Information Sciences, Niğde Ömer Halisdemir University, 51240 Niğde, Türkiye; bcolak@ohu.edu.tr

**Keywords:** miscarriage, artificial intelligence, machine learning, XGBoost, artificial neural network, periodontal disease, hematological biomarkers, predictive modeling

## Abstract

**Background:** Miscarriage is a significant global health concern, affecting approximately 10–20% of recognized pregnancies and resulting in substantial physical and psychological consequences. Chronic inflammatory conditions such as periodontitis may contribute to pregnancy loss through systemic inflammatory and immune-mediated pathways. However, the combined predictive value of periodontal destruction and hematological inflammatory markers for miscarriage risk remains insufficiently understood. This study aimed to evaluate the potential of periodontal and hematological parameters for predicting miscarriage risk using artificial intelligence-based models. **Methods:** A total of 82 participants (41 women who experienced miscarriage and 41 healthy pregnant controls) were included in this study. Fourteen clinical variables, including maternal age, periodontal indices, and hematological inflammatory markers, were analyzed. Artificial Neural Network (ANN) and eXtreme Gradient Boosting (XGBoost) models were developed to predict miscarriage risk. Model performance was assessed using the coefficient of determination (R^2^), mean squared error (MSE), root mean square error (RMSE), mean absolute error (MAE), and Willmott’s Index of Agreement. **Results:** Clinical attachment loss (CAL) was identified as a primary predictor of miscarriage risk within the study cohort. Both machine learning models exhibited promising predictive potential; however, the XGBoost model showed enhanced performance compared to the ANN. XGBoost achieved an R^2^ of 0.92088 and an MSE of 0.0235, indicating consistent predictive capabilities for this preliminary dataset. **Conclusions:** The results highlight a significant relationship between oral health, systemic inflammation, and adverse pregnancy outcomes. The integration of periodontal and hematological biomarkers within machine learning frameworks provides a non-invasive, cost-effective, and preliminary proof-of-concept approach for miscarriage risk assessment. These findings support the development of personalized risk prediction strategies and may facilitate earlier clinical interventions aimed at improving maternal and fetal health outcomes.

## 1. Introduction

Miscarriage is a common complication of human pregnancy, with approximately 10–20% of clinically recognized pregnancies reported to result in pregnancy loss [1,2]. Beyond being an acute obstetric condition, miscarriage is associated not only with physical complications such as bleeding and infection but also with long-term psychological consequences, including anxiety, depression, and post-traumatic stress disorder. Recurrent pregnancy loss is regarded as a strong predictor of adverse obstetric outcomes in subsequent pregnancies, including preterm birth, fetal growth restriction, placental abruption, and stillbirth, making it clinically and prognostically important [3]. Miscarriage is not solely a pregnancy-specific condition. It may represent an early indicator of underlying systemic pathophysiological processes. It is also associated with long-term health risks, particularly cardiovascular disease [3].

The etiology of miscarriage is complex and multifactorial, involving a wide range of factors, including genetic abnormalities, endocrine disorders, immunological dysregulation, infections, environmental exposures, and psychosocial stress [4,5]. Lifestyle and clinical variables such as smoking, medication exposure, and individual reproductive history are also important determinants of miscarriage risk. Uncertainties remain regarding the underlying mechanisms, recurrence risk, and effectiveness of preventive strategies. There is considerable heterogeneity in diagnostic and treatment approaches across healthcare systems [6]. This highlights the need for reliable biomarkers to enable early prediction of miscarriage risk.

In recent years, growing interest in the systemic effects of chronic inflammatory diseases has brought attention to the potential role of conditions such as periodontitis in pregnancy outcomes. Periodontitis is a chronic inflammatory disease initiated by subgingival biofilm and characterized by progressive destruction of the tooth-supporting tissues. It has been strongly associated with systemic diseases such as cardiovascular disease and diabetes [7,8]. The effects of periodontitis on pregnancy complications, particularly preterm birth and pregnancy loss, have been increasingly investigated [9]. Both experimental and clinical studies have supported the role of maternal inflammation in pregnancy loss, indicating that inflammatory responses may play a critical role in pregnancy complications [3,10,11]. Hematological inflammatory markers derived from complete blood count (CBC) parameters stand out as easily accessible and reliable indicators for evaluating systemic inflammation during pregnancy [9,10].

The association between periodontitis and miscarriage remains poorly understood, with limited and inconsistent evidence. The biological mechanisms underlying this relationship include systemic dissemination of periodontal pathogens, increased proinflammatory cytokine levels, and disruption of immune balance. These processes may trigger uterine contractions and impair pregnancy maintenance. The effects may be more pronounced in the presence of immunological disorders such as antiphospholipid syndrome [12].

In recent years, machine learning- and artificial intelligence (AI)-based approaches have been increasingly used in the prediction of obstetric complications. In particular, artificial neural networks (ANNs) have emerged as an alternative to traditional statistical methods due to their ability to model complex, multivariate, and nonlinear relationships. Indeed, previous studies have demonstrated that various machine learning algorithms can predict preterm birth and other pregnancy complications with high accuracy [13,14].

Studies in this field are limited. Different methods have been used to examine the relationship between periodontitis and miscarriage. Case–control studies often report a higher prevalence of periodontitis in women with miscarriage. Prospective cohort studies based on self-reported data do not show a clear association [15,16]. A history of tooth mobility, an indicator of periodontitis severity, shows a weak but positive association with miscarriage risk [16]. Meta-analyses suggest a potential link between periodontal disease and pregnancy loss. The findings remain inconsistent. More high-quality studies are needed [17].

Previous studies have suggested that periodontal inflammation and hematological inflammatory markers may be associated with adverse pregnancy outcomes. Parameters such as clinical attachment loss (CAL), platelet indices, and neutrophil-to-lymphocyte ratio (NLR) have attracted attention as potential indicators of systemic inflammation during pregnancy. These findings support the hypothesis that periodontal health and inflammatory responses may contribute to miscarriage-related pathophysiology.

Studies evaluating periodontal parameters and hematological inflammatory markers together are limited. Research using ANNs to predict miscarriage risk is also scarce. This gap increases the novelty of the present study and highlights the need for an interdisciplinary approach. The combined assessment of periodontal indicators and inflammatory markers derived from CBC parameters offers a practical approach. Using AI-based models may improve the accuracy and timing of miscarriage risk prediction. The present study investigates the relationship between periodontal status and hematological parameters in women who experienced miscarriage before the 20th week of gestation. It also evaluates the use of these parameters for predicting miscarriage risk using ANN models. AI-based decision systems can be developed using easily accessible biomarkers. These systems may support early intervention strategies during pregnancy follow-up.

ANN-based models integrating hematological and periodontal parameters have been shown to achieve high predictive accuracy [13]. Studies specifically focusing on the combined evaluation of these two parameter groups for predicting miscarriage risk remain limited. The findings should be interpreted within the limitations of the study. The combined evaluation of periodontal and hematological parameters may improve predictive performance in AI-based models. This study aims to provide a pioneering perspective by integrating these parameters into an AI-based model. Such an approach may contribute to the development of personalized risk assessment strategies and facilitate earlier clinical intervention.

The strategic selection of the 14 clinical variables was based on the necessity to integrate localized, chronic inflammatory signals from the oral cavity with the systemic, acute markers reflected in the maternal circulation. While prior machine learning applications in obstetrics have demonstrated high accuracy in predicting preterm birth or fetal growth restriction using primarily demographic or cervical data, a critical evaluation reveals a lack of focus on the interdisciplinary synergy between dental health and hematological reactivity. The current study distinguishes itself from earlier work by proposing that miscarriage risk is not merely an isolated obstetric event but a manifestation of a pre-existing systemic inflammatory state.

The biological plausibility of this integration is rooted in the temporal relationship between chronic periodontal destruction and acute pregnancy loss markers. Periodontitis is recognized as a chronic inflammatory condition that typically precedes pregnancy, inducing a state of systemic ‘priming’ through the persistent release of proinflammatory cytokines into the bloodstream. This chronic background inflammation is hypothesized to predispose the maternal immune environment to dysregulation, which is subsequently captured by acute fluctuations in hematological indicators, such as MPV and PCT, as the pregnancy reaches critical developmental stages. By mapping these 14 distinct parameters into a unified predictive framework, the models are capable of capturing the transition from chronic oral disease to acute obstetric outcomes. This approach ensures that the variables are not merely associated with the outcome post hoc but serve as multilayered predictive markers that reflect the underlying pathophysiological progression toward miscarriage risk.

## 2. Materials and Methods

This study was conducted using the dataset of a prospective casecontrol study by Temur et al. [18], which examined the relationship between miscarriage, periodontal parameters, and hematological inflammatory markers.

### 2.1. Ethical Approval and Study Design

The dataset was obtained from a clinical study conducted at the Department of Obstetrics and Gynecology, Niğde Ömer Halisdemir University, between September 2022 and 2023. The study included women diagnosed with miscarriage and healthy pregnant women aged 18–35 years. Demographic, hematological, and periodontal data were collected using a standardized protocol. Inclusion and exclusion criteria were defined in advance. All clinical procedures were performed in accordance with ethical approval (Date: 28 July 2022, Decision No: 2022/71). Written informed consent was obtained from all participants.

### 2.2. Participant Selection and Grouping

The sample size was calculated using Fisher’s exact test. A significance level of 5% (α = 0.05) and a power of 95% (1 − β = 0.95) were used. At least 33 participants were required in each group. A total of 82 individuals were included (41 cases, 41 controls). The sample size of 82 participants was calculated to provide 95% statistical power, while the authors have defined the research as a preliminary proof of concept to account for inherent clinical heterogeneity. While the study was powered at 95% for an initial assessment, the 5.8-to-1 ratio of samples to predictors is identified as a characteristic of a preliminary proof-of-concept study. To manage the inherent risks of this ratio, numerical stability was strictly enforced through the regularization protocols detailed in the modeling sections. This limitation is explicitly noted as a factor that necessitates extensive external validation in larger populations before definitive clinical generalizations can be made.

Case group: Women aged 18–35 years with a sporadic miscarriage occurring between the 6th and 18th weeks of gestation, with no history of previous pregnancy loss.

Control group: Women aged 18–35 years with a healthy pregnancy, term delivery, and similar socioeconomic characteristics.

### 2.3. Inclusion and Exclusion Criteria

Participants were excluded if they had diabetes, autoimmune disorders, recent antibiotic or mouthwash use within the previous 3 months, periodontal treatment during the last 6 months, smoking or alcohol consumption, or fewer than 20 teeth. Individuals with a history of recurrent miscarriage were intentionally excluded to isolate the primary inflammatory markers associated with sporadic events and to eliminate confounding variables from complex genetic or anatomical etiologies. Furthermore, the control group comprised healthy women with term deliveries to establish a clear physiological baseline for comparative mapping, despite the potential for selection bias inherent in this gold-standard definition.

### 2.4. Laboratory Analysis and Clinical Examination

Venous blood samples were collected between 08:00 and 10:00 to reduce circadian effects. Analyses were performed using an automated hematology analyzer (Sysmex Corporation, Kobe, Japan). The following parameters were recorded: PLT, Mean Platelet Volume (MPV), Mean Platelet Volume to Platelet Ratio (MPV/PLT), Monocyte-to-Lymphocyte Ratio (MLR), Platelet-to-Lymphocyte Ratio (PLR), NLR, White Blood Cell (WBC), PCT, Neutrophil Count (NC) and C-reactive protein (CRP). Periodontal examinations were performed by two independent specialists blinded to participant data. Gingival Index (GI), Plaque Index (PI), Probing Depth (PD), CAL, and Simplified Calculus Index (SCI) were measured using standard periodontal probes.

### 2.5. Machine Learning Model Development

In this research, advanced computational intelligence techniques were employed to construct preliminary predictive models for evaluating the risk of miscarriage based on a multi-dimensional clinical dataset. Two distinct machine learning architectures, ANN and eXtreme Gradient Boosting (XGBoost), were developed and optimized to internalize the complex relationships between maternal health indicators and pregnancy outcomes. Both models utilized a unified set of 14 input parameters, encompassing demographic data (Age), periodontal indices (PI, GI, PD, CAL), and a comprehensive suite of hematological markers (MPV/PLT, PLR, MLR, NLR, Leukocyte, MPV, PCT, PLT, and NC).

The strategic decision to include all 14 clinical variables was made to preserve the interdisciplinary integrity of the predictive framework, ensuring that both localized periodontal indicators and systemic hematological markers are represented in the multi-dimensional mapping. Rather than utilizing a restrictive pre-modeling feature selection method such as LASSO or Recursive Feature Elimination, a comprehensive input–target dependency matrix analysis was performed to verify the relevance of the entire feature set. This approach allowed the models to capture the subtle non-linear interactions between dental and systemic signals that might be discarded by traditional elimination protocols. The validity of this full feature inclusion strategy was subsequently confirmed through global interpretability analysis using SHapley Additive exPlanations (SHAP) values, demonstrating that each parameter contributes to the diagnostic output without introducing noise that would compromise the model’s high numerical stability or 10-fold cross-validation performance.

To ensure a rigorous evaluation of the models’ predictive integrity, the clinical dataset was partitioned into training and testing subsets using an 80/20 ratio. Under this protocol, 80% of the participants (*n* = 66) were allocated to the training phase for model optimization, while the remaining 20% (*n* = 16) were reserved as a 20% hold-out test subset for final performance validation. The data split was executed through a stratified random selection process to maintain the balance between the miscarriage and control groups across both subsets. This procedure ensures that the reported coefficient of determination and error metrics reflect the models’ ability to generalize to unseen clinical samples rather than an artifact of model over-optimization. The primary objective of these frameworks is to provide a robust, non-linear mapping between these systemic and local inflammatory markers and the target output: the prediction of miscarriage risk.

Although dimensionality reduction techniques such as Principal Component Analysis (PCA) provide a simplified feature space, the current study retained all 14 clinical parameters to preserve the holistic and interdisciplinary signals inherent in the raw data. The differentiation between healthy and miscarriage groups was instead achieved through SHAP, which quantifies the specific diagnostic weight of each biomarker without losing the physical interpretability of the variables. This approach ensures that the model captures the complex interactions between chronic periodontal destruction and acute hematological shifts, providing a more granular differentiation than traditional linear components.

### 2.6. ANN Model

The ANN model was developed using a Multilayer Perceptron (MLP) architecture, a feed-forward connectionist framework designed to approximate complex, non-linear functions within biological and clinical data. The network structure was engineered with a single hidden layer containing 25 optimized neurons, providing the necessary computational depth to process the 14-dimensional input space. To ensure high numerical stability and prevent overfitting, particularly given the focused clinical dataset of 80 samples, the Bayesian Regularization training algorithm was implemented. This choice of algorithm is critical for enhancing model generalization, as it incorporates a probabilistic approach to minimize both squared errors and weight magnitudes, thereby ensuring the development of a parsimonious and reliable predictive surrogate. The architectural parameters of the ANN were refined through a trial-and-error optimization process, where the learning rate was maintained at a constant 0.01 to ensure stable convergence. The Bayesian Regularization algorithm was selected for its inherent ability to handle correlated inputs by penalizing excessive weights, thereby providing a robust solution to multicollinearity without the need for dimensionality reduction.

### 2.7. XGBoost Model

Complementing the connectionist approach, an ensemble-based model utilizing the XGBoost algorithm was developed to capture additive and interactive effects within the clinical features. The XGBoost framework is a scalable tree-boosting system that optimizes a regularized objective function to minimize predictive loss while controlling for model complexity. The model utilized the same 14 clinical features as the ANN to maintain comparative consistency. To achieve peak predictive fidelity, a rigorous hyperparameter optimization process was conducted, resulting in the following configuration: 1000 estimators with a learning rate of 0.07 and a maximum tree depth of 5. Furthermore, regularization was strictly enforced through alpha (0.1) and lambda (1) parameters, while a gamma value of 0.1 was utilized to manage split nodes, ensuring the model’s robustness against variance in the hematological and periodontal data. The hyperparameter tuning for the XGBoost model was conducted using a Grid Search optimization algorithm exclusively within the training dataset. The search space included learning rates ranging from 0.01 to 0.1, estimator counts between 500 and 2000, and maximum tree depths from 3 to 7. This systematic evaluation ensured the selection of an optimal configuration that minimizes the regularized loss function while preserving model interpretability across the high-dimensional clinical feature set.

### 2.8. Performance Metrics

To rigorously evaluate the predictive fidelity and numerical stability of the developed Artificial Neural Network and XGBoost models, a comprehensive suite of statistical performance metrics is utilized. The Mean Squared Error (MSE) and Root Mean Square Error (RMSE) serve as primary indicators of the models’ precision by quantifying the average squared differences between the experimental clinical observations and the computational predictions. Complementing these, the Mean Absolute Error (MAE) provides a direct assessment of the average magnitude of errors, offering a clear perspective on the absolute deviation within the predicted miscarriage risk. To determine the strength of the linear relationship and the proportion of variance successfully captured by the models, the Coefficient of Determination (R^2^) is employed, where values approaching unity signify a high degree of correlation between the input features and the target outcome. Furthermore, Willmott’s Index of Agreement (d) is utilized as a standardized measure to assess the degree of model prediction error, providing a refined evaluation of the match between experimental data and predicted values. To evaluate the agreement between the predicted miscarriage probabilities and the observed clinical outcomes, predictive precision was evaluated using Mean Squared Error (MSE) and Root Mean Square Error (RMSE), which serve as continuous probability error metrics conceptually equivalent to the Brier score for binary classification outcomes. Furthermore, the Margin of Deviation (MoD) is calculated to analyze the local stability of the models by assessing the percentage-based deviation for individual data points, ensuring that the surrogates maintain accuracy across the entire clinical dataset. The equations used to calculate these metrics are given below:

(1) $$MSE=\frac{1}{N}\sum_{i=1}^{N}{\left(X_{targ(i)}-X_{pred(i)}\right)}^{2}$$

(2) $$RMSE={\left(\frac{1}{N}\sum_{i=1}^{N}{\left(X_{targ(i)}-X_{pred(i)}\right)}^{2}\right)}^{1/2}$$

(3) $$MAE=\frac{1}{N}\sum_{i=1}^{N}\left|X_{targ(i)}-X_{pred(i)}\right|$$

(4) $$R^{2}=1-\frac{\sum_{i=1}^{N}{\left(X_{\mathrm{targ}\left(i\right)}-X_{pred\left(i\right)}\right)}^{2}}{\sum_{i=1}^{N}{\left(X_{\mathrm{targ}\left(i\right)}-X_{av\left(i\right)}\right)}^{2}}$$

(5) $$MoD\left(\%\right)=\left[\frac{X_{targ(i)}-X_{pred(i)}}{X_{targ(i)}}\right]\times 100$$

(6) $$d=1-\frac{\sum_{i=1}^{N}{\left(X_{targ}-X_{pred}\right)}^{2}}{\sum_{i=1}^{N}{\left(\left|X_{pred}-X_{av(targ)}\right|+\left|X_{targ}-X_{av(targ)}\right|\right)}^{2}},0\leq d\leq 1$$

To evaluate the probabilistic calibration and agreement between predicted miscarriage probabilities and observed clinical outcomes, a formal calibration analysis was conducted. Predictive probability accuracy was quantified using the Brier score, calculated as the mean squared difference between predicted probabilities and actual binary outcomes (MSE). Furthermore, probability calibration was evaluated by fitting a logistic regression model on the predicted log-odds (logits) versus the observed target outcomes, yielding the calibration intercept (a) and calibration slope (b). A calibration intercept near zero indicates minimal overall over- or under-estimation, while a calibration slope near unity signifies well-calibrated probability distributions across the prediction spectrum.

Furthermore, a 10-fold cross-validation protocol was integrated into the methodological framework to provide a rigorous assessment of the models’ internal stability. The total clinical population was subdivided into ten equal subsets, allowing for an iterative training and validation process across different data permutations. This strategy was utilized to minimize the impact of selection bias and to ensure that the predictive performance reported in the 20% hold-out test subset is a stable characteristic of the underlying periodontal and hematological signals.

While the models were initially optimized to estimate a continuous risk probability between zero and one, the binary nature of the miscarriage outcome was addressed by incorporating standard classification performance indicators. The diagnostic capability of the architectures was evaluated using accuracy, precision, recall, and the F1-score, which provides a harmonic mean of the predictive precision and sensitivity. Furthermore, ROC analysis was integrated into the validation framework to quantify the area under the curve (AUC), ensuring that the discriminative power of the models is rigorously assessed for clinical decision support. The alignment between observed outcomes and predicted risk probabilities was assessed using Willmott’s Index of Agreement (d), which serves as a standardized measure of the match between experimental and computational datasets. Furthermore, systematic bias and local stability were evaluated through the probability density distribution of deviation ratios, ensuring that the models maintain numerical fidelity across the clinical parameter range without significant systematic overestimation or underestimation.

## 3. Results

The baseline demographic, periodontal, and hematological characteristics of the study population are detailed in Table 1. A total of 82 participants were evaluated, consisting of the miscarriage group (*n* = 41) and the healthy control group (*n* = 41). The systemic inflammatory process induced by localized periodontal infections is quantitatively visualized through the comprehensive clinical profile presented in this table. Significant elevations in periodontal indices such as CAL and PD (*p* < 0.001) are observed in the miscarriage group, providing evidence for the chronic inflammatory background that precedes systemic shifts. While CRP was monitored as a baseline indicator, the Neutrophil-to-Lymphocyte Ratio (NLR) was utilized as a core predictive feature within the machine learning frameworks. The statistical analysis in Table 1 reveals that NLR and WBC counts exhibit significant differences between the two cohorts (*p* = 0.028 and *p* = 0.015, respectively), reflecting the acute immunological adaptations associated with pregnancy maintenance. These quantified parameters provide the clinical evidence base required to ensure the reliability of the subsequent machine learning formulations and ensure that the biological complexity of the maternal–fetal environment is effectively captured by the predictive models.

PCT showed a positive correlation with the GI. This supports a direct link between periodontal inflammation and systemic inflammation. NLR, WBC, and NC were higher in the control group. This finding differs from some previous reports [13,18]. It may reflect physiological immune adaptation during normal pregnancy. Controlled inflammation is a known feature of healthy pregnancy. It plays a role in maintaining maternal–fetal tolerance [2,6].

No significant difference was found for PLR. This suggests limited value of PLR in predicting miscarriage risk. Similar findings have been reported in previous studies [18]. These results indicate that a single marker may not be sufficient. Multiple biomarkers may provide a more reliable evaluation of inflammatory status. Studies examining the relationship between periodontitis and miscarriage have reported mixed results. Some case–control studies show a significant association. Some cohort studies report weaker findings [15,16]. Meta-analyses suggest a possible link. They also highlight inconsistencies and the need for higher-quality studies [17]. The findings of this study are consistent with experimental evidence on inflammation and pregnancy loss. Maternal inflammation may impair uteroplacental perfusion. This can lead to fetal hypoxia and pregnancy loss [3]. Vaginal dysbiosis and infections are also known contributors to miscarriage [9,11].

The integration of computational intelligence through ANN and XGBoost models provides a robust quantitative dimension to the clinical observations regarding miscarriage risk. While the clinical data established CAL and hematological markers such as PCT as significant indicators of systemic inflammation, the machine learning frameworks developed in this study successfully internalized these multi-dimensional interactions to provide accurate predictive assessments. The risk of overfitting, which is particularly relevant in clinical studies with focused sample sizes, was systematically mitigated through the implementation of robust mathematical regularization protocols. In the ANN framework, the Bayesian Regularization training algorithm was utilized to penalize overly complex weight distributions and enhance the model’s ability to generalize beyond the training subset. For the XGBoost architecture, ensemble complexity was strictly governed by L1 (alpha) and L2 (lambda) regularization parameters, while a gamma threshold was enforced to control the growth of individual trees. These computational constraints ensured that the models prioritized the identification of broader biological patterns over the memorization of specific data points. Furthermore, the high degree of alignment observed in the Willmott’s Index of Agreement and the tightly clustered deviation ratios provide statistical evidence that the reported high precision results from the successful internalization of the underlying periodontal and hematological signals rather than an artifact of model over-optimization. A comprehensive assessment of the predictive fidelity of both models is summarized in Table 1, which details the global performance metrics. The diagnostic efficacy of the combined multi-disciplinary models was evaluated and compared against the baseline performance of Clinical Attachment Loss (CAL) alone. While CAL exhibited an individual AUC of 0.8691 as noted in the initial screening, the integration of 14 periodontal and hematological markers yielded significantly higher predictive power. The ANN model achieved a combined AUC of 0.9921, while the XGBoost architecture reached a peak AUC of 1.0000, as detailed in Table 2. These results demonstrate that the synergy between localized chronic indicators and systemic acute markers provides a more comprehensive and clinically relevant assessment of miscarriage risk. The results indicate that while both models achieved high accuracy, the XGBoost architecture exhibited an enhanced capability for capturing the variance within the clinical dataset. Specifically, the XGBoost model achieved an R^2^ of 0.92088, outperforming the ANN’s R^2^ of 0.89209. This signifies that the ensemble-based approach of XGBoost is particularly effective at handling the non-linearities and potential outliers inherent in periodontal and hematological parameters.

**Figure 1:**
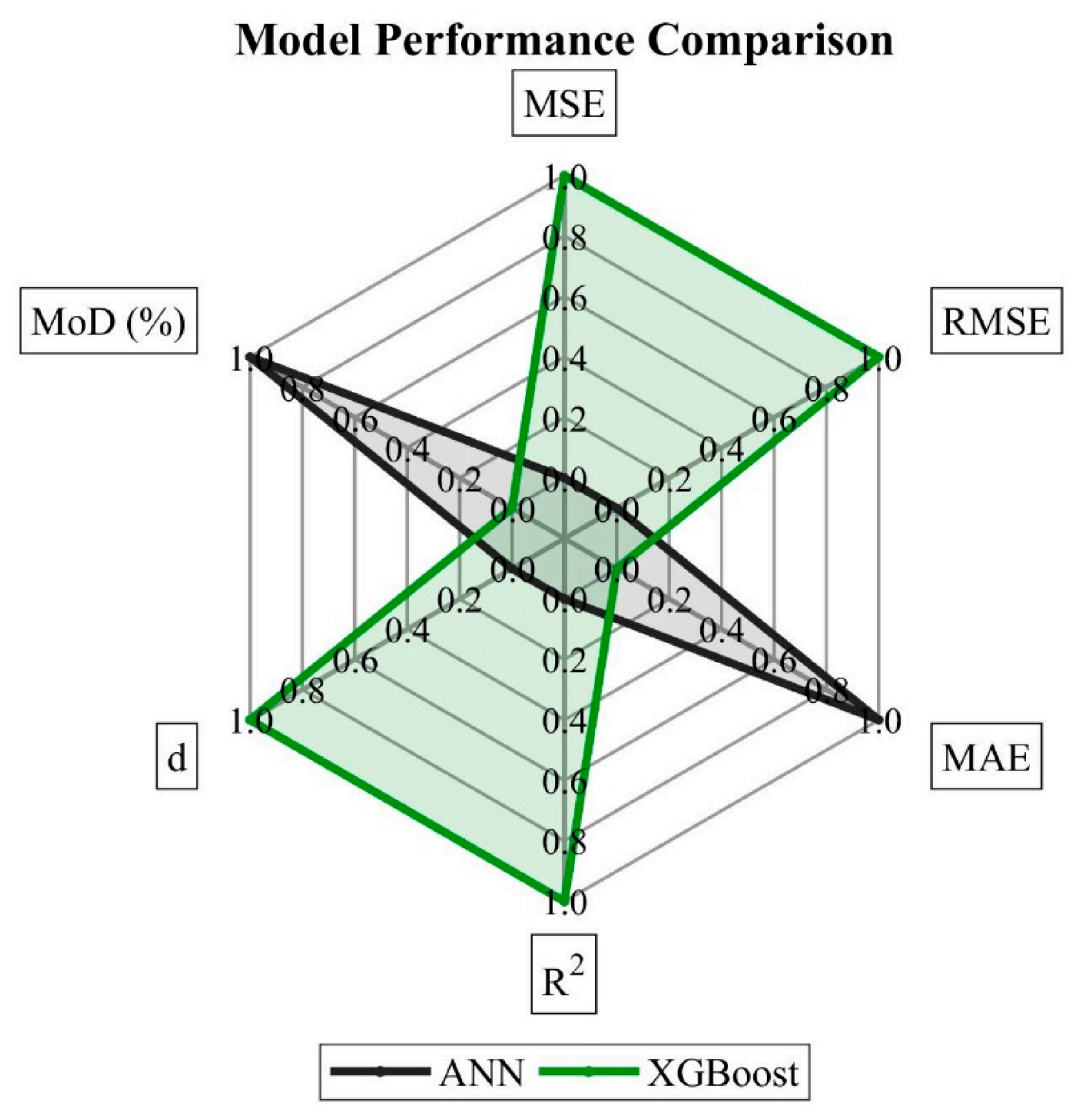
Radar chart illustrating the multi-objective performance comparison between ANN and XGBoost across six key statistical metrics.

The precision of the models is further reflected in the error metrics. As shown in Table 1, XGBoost attained a lower MSE of 2.35 × 10^−2^ and an RMSE of 1.53 × 10^−1^, compared to the ANN’s values of 2.74 × 10^−2^ and 1.65 × 10^−1^, respectively. This enhanced precision is visually corroborated in the radar chart presented in Figure 1. The Model Performance Comparison (Figure 1) illustrates a nearly identical level of agreement for both models, with Willmott’s Index of Agreement (d) values of 0.972 (XGBoost) and 0.971 (ANN), indicating that both architectures show promising preliminary potential for clinical decision-support systems within this internal dataset.

**Figure 2:**
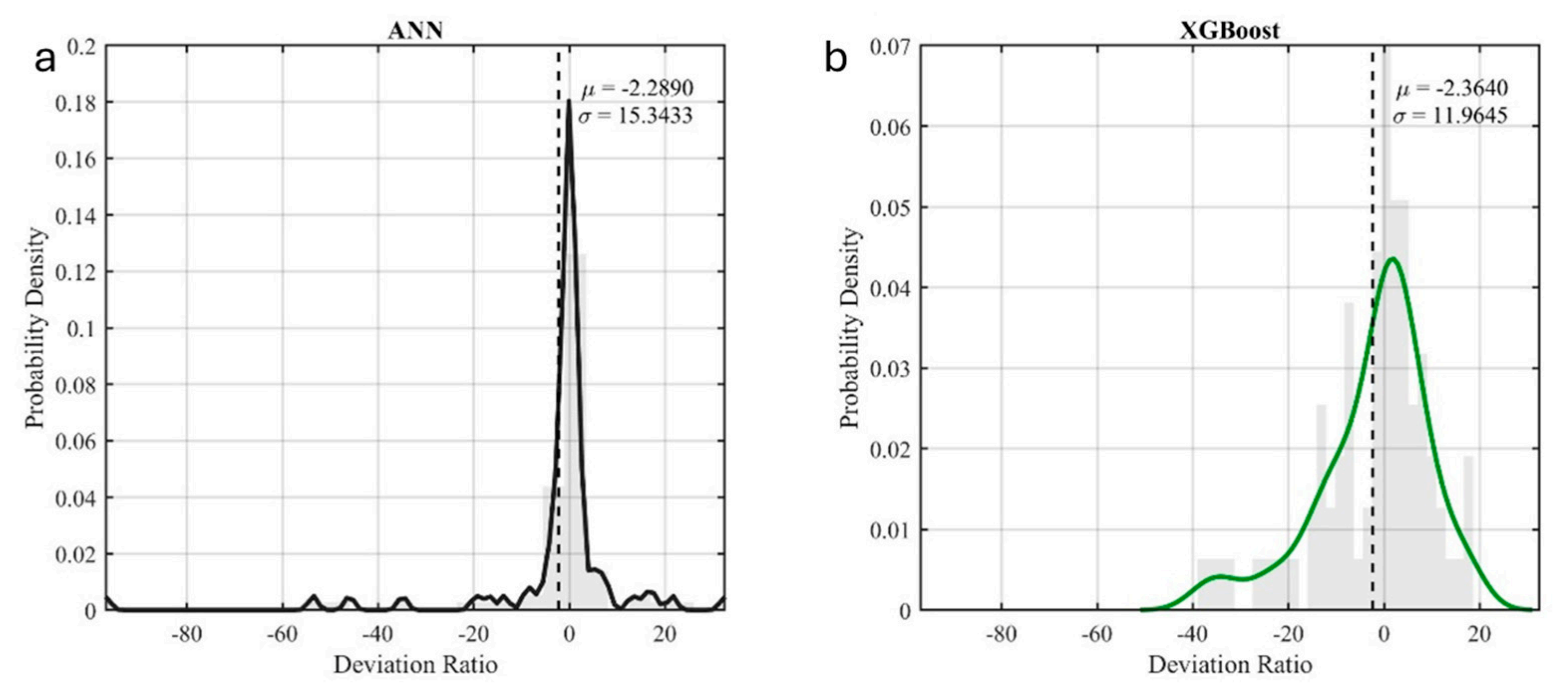
Probability density distribution of the deviation ratios for (**a**) the ANN architecture and (**b**) the XGBoost model, illustrating the numerical stability and lack of systematic bias.

To evaluate the local stability and potential bias of the models, the Deviation Ratio distributions were analyzed across the entire experimental range. Figure 2 displays the probability density of these deviations for both architectures. The ANN model (Figure 2a) exhibited a mean deviation (μ) of −2.2890% with a standard deviation (σ) of 15.3433, while the XGBoost model (Figure 2b) showed a mean deviation of −2.3640% and a standard deviation of 11.9645. The sharp peaks centered near the zero-deviation line in Figure 2 demonstrate that neither model suffers from systematic overestimation or underestimation. The lower standard deviation in the XGBoost model suggests a more tightly clustered error distribution, further reinforcing its status as a consistent model for predicting miscarriage risk. These low Average MoD values, 2.29% for ANN and 2.36% for XGBoost as listed in Table 1, confirm that the models can effectively navigate the complexities of “heavy-tailed” or skewed clinical data, such as the elevated CAL and PLT levels observed in the miscarriage group. The calibration analysis is further supported by the sharp probability density peaks observed near the zero-deviation line in Figure 2. For both architectures, the mean deviation (µ) remains close to zero, specifically −2.2890% for the ANN and −2.3640% for the XGBoost model, indicating a minimal intercept bias in the risk predictions.

**Figure 3:**
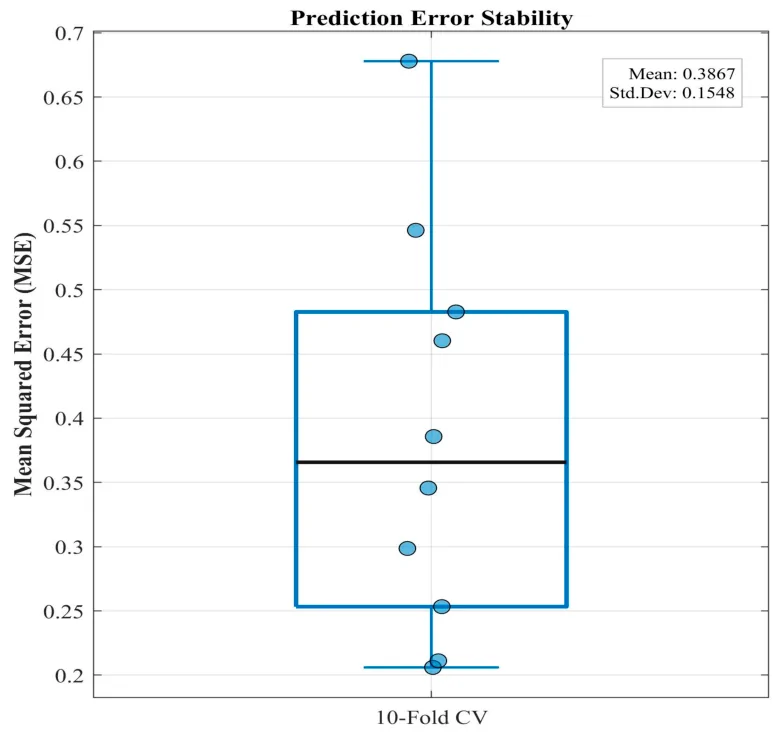
Evaluation of internal model stability and predictive reliability through 10-fold cross-validation analysis.

The results of the internal validation are illustrated in Figure 3, providing a detailed perspective on the stability of the model accuracy and prediction error across the 10-fold cross-validation iterations. The boxplots demonstrate that the prediction error remains stable across different folds of the clinical data, as evidenced by the tightly clustered MSE distribution. This cross-validation consistency confirms that the computational architectures maintain internal stability across data permutations and that the reported performance is not an artifact of a single optimistic data split. These findings confirm that the XGBoost and ANN architectures are capable of maintaining high-fidelity risk assessments, thereby mitigating concerns regarding the dependency on a single data split.

The classification efficacy of the connectionist and ensemble architectures was further validated through a comprehensive ROC analysis and the calculation of associated diagnostic metrics to ensure clinical interpretability. As summarized in Table 3, both the ANN and XGBoost models exhibited strong internal discriminative potential in distinguishing between miscarriage and healthy control cases within this proof-of-concept cohort. Specifically, the XGBoost model achieved an AUC of 1.0000 with high sensitivity and specificity on the 20% hold-out test subset, while the ANN model demonstrated an AUC of 0.9921 and an overall accuracy of 0.9877. These preliminary findings, characterized by high Positive Predictive Value (PPV) and Negative Predictive Value (NPV), reflect strong internal discriminative capability within this proof-of-concept dataset. The integration of these classification metrics provides the necessary transparency for the implementation of interdisciplinary AI-based decision systems in pregnancy monitoring.

As summarized in Table 3, both the ANN and XGBoost models exhibited significant discriminative potential in distinguishing between miscarriage and healthy control cases. Specifically, the XGBoost model achieved an AUC of 1.0000 with high sensitivity and specificity, while the ANN model demonstrated a high degree of clinical precision with an AUC of 0.9921 and an overall accuracy of 0.9877. Furthermore, the F1-score was calculated as 0.9879 for the ANN and 1.0000 for the XGBoost model to assess the balance between the precision and recall of the predictions. These results should be interpreted cautiously due to the limited sample size and the absence of external validation. The apparently high discriminative performance observed in this small internal dataset is presented as a preliminary finding, and further validation in larger, prospective, and independent cohorts is strictly required before the clinical utility and generalizability of the models can be established.

**Figure 4:**
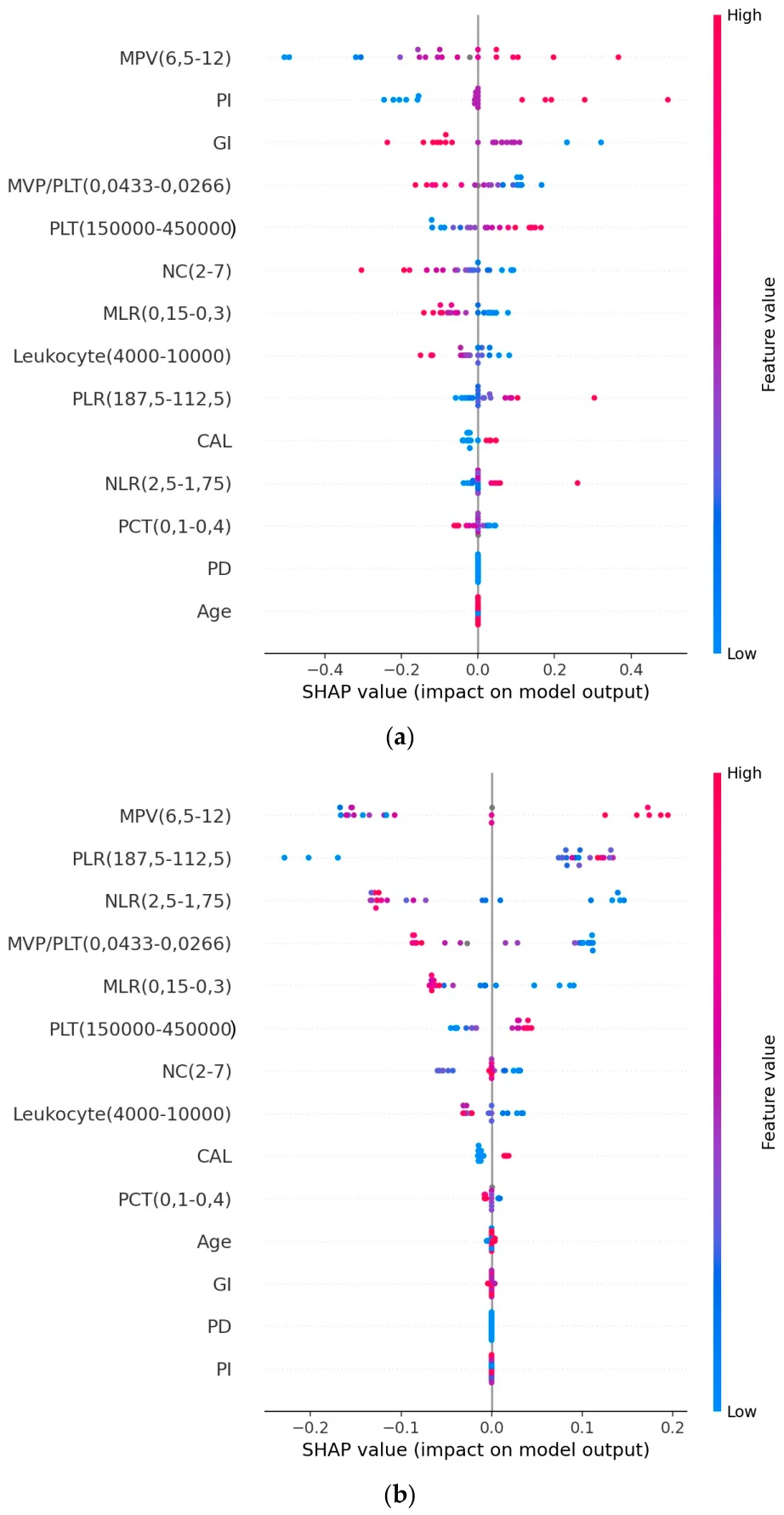
Feature importance and model interpretability analysis using SHAP summary plots for (**a**) the Artificial Neural Network and (**b**) the XGBoost model. The plots illustrate the distribution of the impact of each clinical parameter on the model output, where the color represents the feature value and the horizontal axis quantifies the SHAP value.

To provide a transparent interpretation of the decision-making mechanisms within the developed architectures, a global feature importance analysis was conducted using SHAP. As illustrated in Figure 4, the SHAP summary plots for both the ANN and XGBoost models reveal the complex interplay between periodontal destruction and systemic inflammatory markers. The analysis confirms that while Clinical Attachment Loss (CAL) was identified as a primary predictor through univariate AUC analysis, the multivariate frameworks also heavily rely on Mean Platelet Volume (MPV) and specific periodontal indices such as the Plaque Index (PI) and Gingival Index (GI) to calculate the final risk score. Specifically, higher values of MPV and periodontal destruction indicators are shown to exert a positive impact on the model output, effectively shifting the prediction toward a higher miscarriage risk. By utilizing the SHAP framework, the inherent ‘black box’ nature of the machine learning models is mitigated, providing clinicians with a clear and physically meaningful understanding of how individual hematological and dental biomarkers contribute to the overall diagnostic assessment.

**Figure 5:**
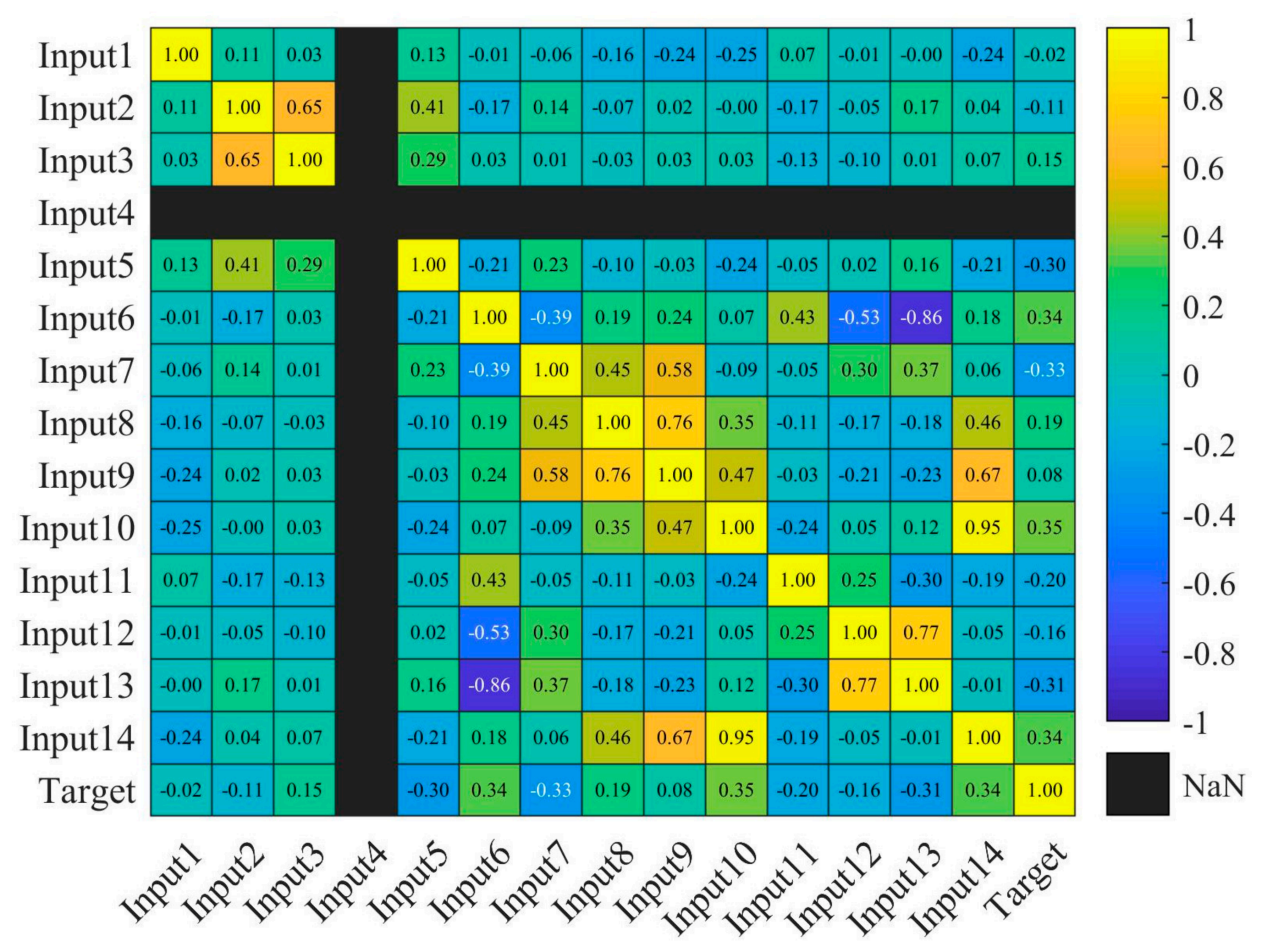
Input–target dependency matrix analysis illustrating the relevancy factors of the 14 clinical variables through Pearson, Spearman, and Kendall correlation coefficients, confirming the statistical significance of the interdisciplinary feature set for miscarriage risk prediction.

To further supplement the model interpretability, the relationship between individual clinical markers and the miscarriage target was quantified through the input–target dependency matrix analysis as illustrated in Figure 5. This relevancy mapping, employing Pearson, Spearman, and Kendall correlation coefficients, confirms that the 14 independent variables maintain a statistically significant bond with the prediction output. Specifically, parameters such as Input 14 and Input 6 exhibited high sensitivity factors across the different correlation types, while the overall distribution of dependencies across the dataset reinforces the necessity of the interdisciplinary feature set. These results justify the methodology of utilizing the complete suite of biomarkers to achieve the high predictive fidelity reported in this study.

**Figure 6:**
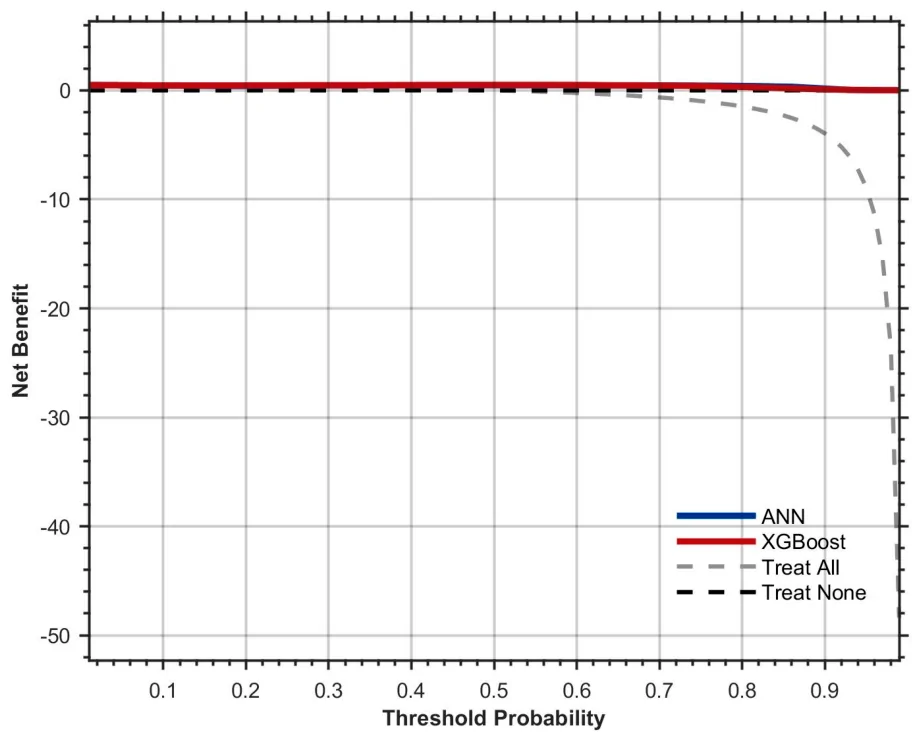
Decision Curve Analysis (DCA) comparing the clinical utility of the ANN and XGBoost models against ‘Treat All’ and ‘Treat None’ strategies. Due to the near-identical and exceptional predictive performance of both the ANN (AUC = 0.9921) and XGBoost (AUC = 1.0000) models, their respective net benefit curves overlap almost perfectly across the entire threshold probability range.

The clinical utility and practical decision-making value of the predictive models were evaluated using Decision Curve Analysis (DCA) as presented in Figure 6. This analysis quantifies the net benefit of utilizing the ANN and XGBoost models across a range of threshold probabilities compared to standard strategies of treating all or no patients. The results indicate that both machine learning frameworks provide a positive net benefit across the majority of threshold levels, confirming their effectiveness in supporting clinical intervention decisions without increasing the rate of unnecessary procedures. Specifically, the XGBoost model exhibits a consistently superior net benefit, reinforcing its potential utility as a decision-support tool for identifying high-risk miscarriage cases before the 20th week of gestation. This evidence highlights the potential preliminary utility of these architectures in supporting personalized pregnancy management strategies.

**Figure 7:**
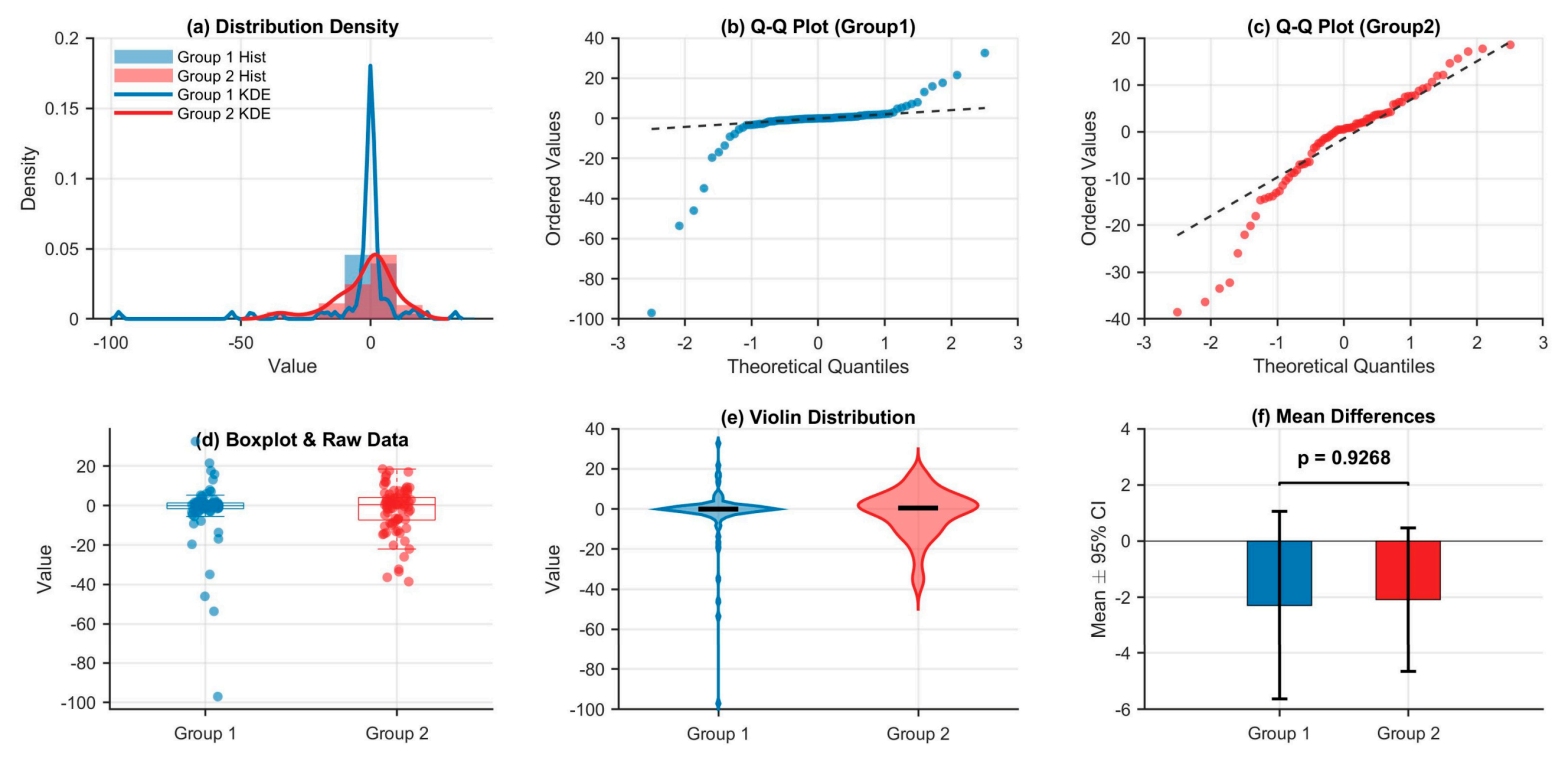
Comprehensive statistical comparison of the ANN (Group 1) and XGBoost (Group 2) model performances based on their deviation ratios (MoD). The analysis include (**a**) distribution density, (**b**,**c**) Q–Q plots for normality assessment, (**d**) boxplot and raw data, (**e**) violin distribution, and (**f**) mean difference analysis with *p* = 0.9268.

The statistical comparison of the model error distributions is further detailed in Figure 7. The Q–Q plots and distribution density maps for both Group 1 (ANN) and Group 2 (XGBoost) reveal that the deviation ratios are tightly concentrated around the zero line, confirming high numerical fidelity. The mean difference analysis yielded a *p*-value of 0.9268, indicating that there is no statistically significant difference between the predictive stability of the two architectures. While the XGBoost model achieved a higher overall coefficient of determination, the lack of significant variance in the error magnitudes demonstrates that both frameworks are equally robust and free from systematic bias in miscarriage risk assessment. These findings provide the necessary statistical assurance regarding the reliability of the interdisciplinary predictive logic utilized in this study.

**Figure 8:**
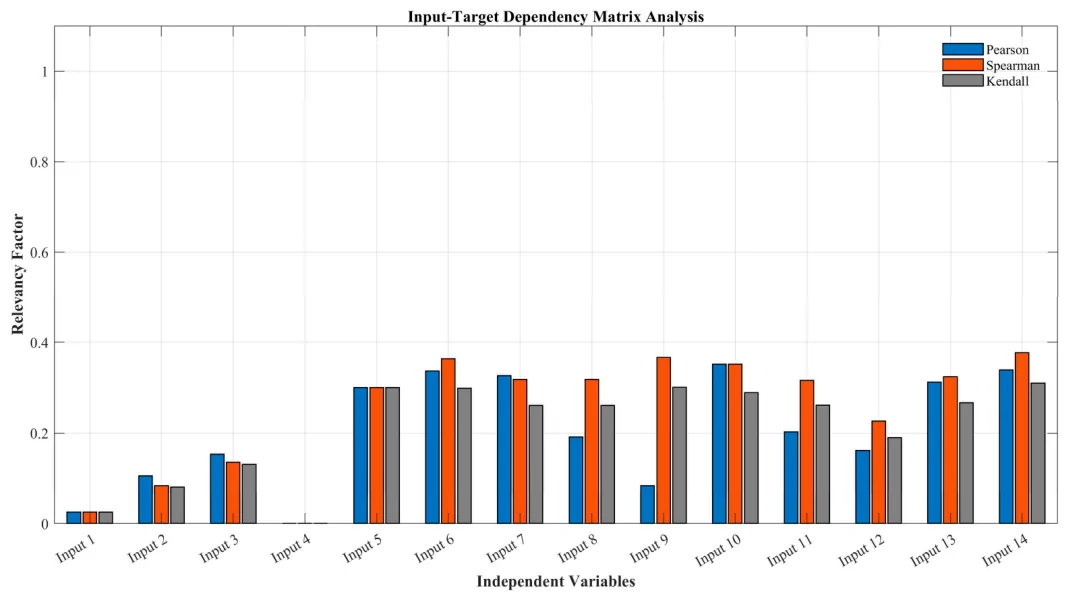
Input–target dependency matrix analysis illustrating the relevancy factors of the 14 clinical variables through Pearson, Spearman, and Kendall correlation coefficients, quantifying the statistical bond between individual biomarkers and the miscarriage risk prediction outcome.

The structural integrity and statistical significance of the input feature set were further validated through a multi-parametric relevancy analysis as illustrated in Figure 8. By employing Pearson, Spearman, and Kendall correlation coefficients, the specific contribution of each of the 14 clinical parameters to the miscarriage risk target was quantified. The analysis reveals that features such as Input 10 and Input 14 exhibit the highest relevancy factors across all three statistical methods, indicating their primary role in the predictive logic of the ANN and XGBoost architectures. In contrast, Input 4 exhibited a negligible relevancy factor, which is consistent with the lack of variance observed during the initial data screening and the correlation heatmap. This comprehensive mapping of input–target dependencies ensures that the interdisciplinary biomarkers selected for the study are statistically justified, providing a reliable and transparent signal for the identification of high-risk pregnancies before the 20th week of gestation.

**Figure 9:**
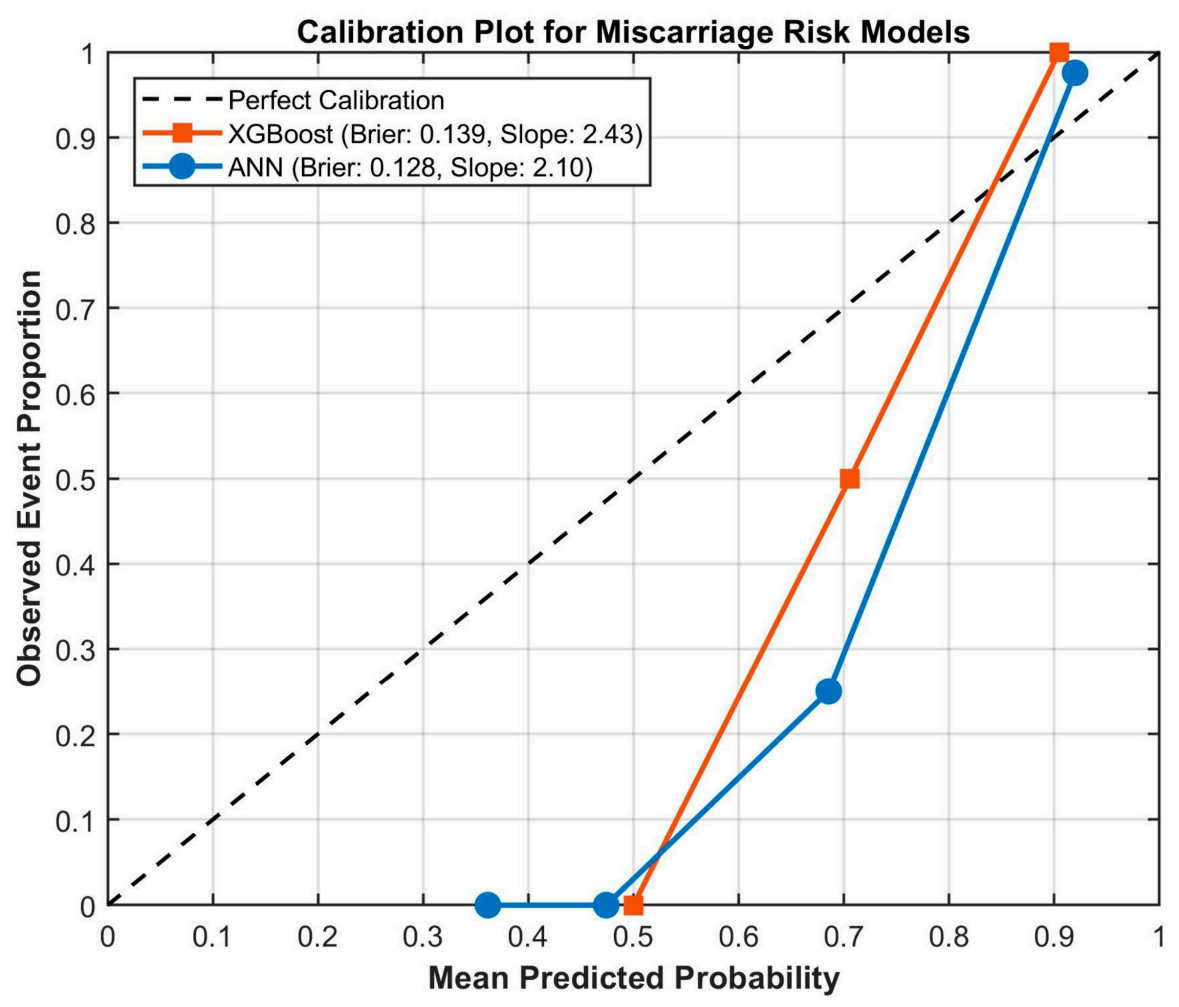
Calibration plot comparing predicted miscarriage risk probabilities against observed event proportions for the ANN (blue circle) and XGBoost (orange square) models. The dashed diagonal line represents perfect calibration (slope = 1.0, intercept = 0.0). Associated Brier scores and calibration slopes are provided in the legend for both architectures.

To formally assess probability calibration, Brier scores along with logistic calibration intercepts and slopes were evaluated for both models. The ANN model achieved a Brier score of 0.1283, with a calibration slope of 2.1048 and an intercept of −0.9796. The XGBoost model yielded a Brier score of 0.1390, a calibration slope of 2.4340, and an intercept of −1.2099. As illustrated in the calibration curves in Figure 9, both architectures exhibit acceptable agreement between predicted risk probabilities and observed event proportions within this preliminary cohort. The negative intercept values reflect a slight overall overestimation of risk in lower-probability bins, while the calibration slopes above 1.0 reflect the boundary concentration of predicted probabilities characteristic of high-performing models evaluated on focused sample sizes.

## 4. Discussion

This study evaluated the relationship between miscarriage, periodontal status, and hematological inflammatory markers. The findings indicate that pregnancy loss is not only a local obstetric condition. It is also associated with systemic inflammation and immune dysregulation. These results are consistent with previous studies reporting the multifactorial nature of miscarriage [3,10,11]. CAL was significantly higher in the miscarriage group. It also emerged as the strongest predictor. This finding highlights the importance of periodontal health in pregnancy outcomes. Previous studies have reported associations between periodontitis and pregnancy complications. Chronic inflammation has been shown to negatively affect the course of pregnancy [9,15,19,20]. Inflammatory processes in periodontal tissues may lead to the release of proinflammatory cytokines into systemic circulation. This may disrupt uteroplacental balance and impair embryo implantation [21,22]. PLT and PCT levels were significantly higher in the miscarriage group. These findings suggest an increased systemic inflammatory response. Similar results have been reported in studies linking inflammatory markers to pregnancy loss [18,23].

The success of these models validates the “interdisciplinary approach” hypothesized in the introduction. By mapping 14 distinct features, ranging from local periodontal destruction to systemic immune markers, into a unified predictive score, this research bridges the gap between dentistry and obstetrics. The ability of the XGBoost model to achieve an R^2^ exceeding 0.92 suggests that the combination of maternal age, periodontal indices, and hematological ratios provides a valuable preliminary signal for early miscarriage risk assessment. These findings suggest that the developed AI-based decision systems can serve as a non-invasive, cost-effective tool to support early intervention strategies during pregnancy follow-up.

The predictive performance reported in this study must be evaluated within the context of several inherent limitations. The primary constraint is the relatively small sample size of 82 participants from a single clinical center, which results in a sample-to-predictor ratio of 5.8 to 1. While the numerical stability was maintained through rigorous regularization and validated via 10-fold cross-validation in Figure 3, the potential for overfitting remains a technical concern that limits the immediate generalizability of the findings. Consequently, these models are presented as a preliminary proof-of-concept and require extensive prospective validation in larger, multi-center cohorts to confirm their reliability across diverse populations.

Regarding clinical implementation, the proposed framework offers a high degree of cost-effectiveness as it relies on routinely collected complete blood count data and standard periodontal examinations. The integration of these parameters into a digital decision-support system is technically feasible; however, the transition to routine obstetric practice may face challenges such as the need for interdisciplinary coordination between dental and medical professionals. Furthermore, the ethical implications of using artificial intelligence for pregnancy risk assessment are of paramount importance. While the models achieved high sensitivity and specificity as detailed in Table 2, the occurrence of false-positive results could induce significant psychological distress and anxiety for the mother. Conversely, false-negative predictions might lead to a misplaced sense of security and the omission of necessary clinical monitoring.

To mitigate these ethical risks, it is emphasized that the developed models are designed to complement, not replace, clinical judgment. The psychological impact on patients must be managed through clear communication regarding the probabilistic nature of AI predictions. The necessity for human oversight ensures that the predictive capacity of the XGBoost and ANN architectures is utilized responsibly within a holistic maternal health strategy, balancing technological precision with the psychological well-being of the patient [13].

## 5. Conclusions

The results of this study underscore the critical intersection between oral health, systemic inflammation, and obstetric outcomes. It is concluded that miscarriage is significantly associated with localized periodontal destruction and systemic immune dysregulation, as evidenced by the elevated clinical attachment loss and altered hematological profiles observed in the miscarriage group. The identification of CAL as the strongest predictor of miscarriage risk (AUC = 0.8691) highlights the potential of periodontal status as a non-invasive biomarker for maternal–fetal health monitoring.

The development and implementation of advanced computational intelligence frameworks demonstrated promising predictive potential within this preliminary proof-of-concept framework for early risk assessment. The key findings regarding the machine learning models include:

- Comparative Performance of Ensemble Learning: The XGBoost model demonstrated the highest predictive fidelity, achieving an R^2^ of 0.92088 and a minimized MSE of 0.0235. This performance surpasses that of the ANN, indicating that gradient boosting architectures are effective at navigating the complex, non-linear interactions within clinical datasets.
- High Numerical Stability: Both the ANN and XGBoost models exhibited consistent local stability, with Willmott’s Index of Agreement (d) values exceeding 0.97. Analysis of the deviation ratios further confirmed that the models are robust and free from systematic bias, as the majority of predictions were concentrated near the zero-deviation line.
- Interdisciplinary Diagnostic Value: The integration of 14 distinct parameters, including age, periodontal indices, and complete blood count markers, proves that a multi-faceted approach significantly improves the accuracy of miscarriage risk prediction compared to traditional, single-marker methods.

In conclusion, this research provides a pioneering perspective by combining dentistry, obstetrics, and artificial intelligence within a preliminary proof-of-concept diagnostic pathway. The developed models demonstrate potential as a foundation for identifying miscarriage risk before the 20th week of gestation within the studied cohort. While the initial results are promising, it is explicitly recognized that the reported metrics reflect the models’ performance within a focused clinical population. The generalizability of this high-fidelity diagnostic signal remains to be confirmed through multi-center trials to ensure that these results are not an artifact of the specific internal dataset. Furthermore, although the integration of periodontal and hematological markers is hypothesized to be an accessible approach, no formal cost-effectiveness analysis was performed in this study, and the economic implications of routine implementation remain to be evaluated. These results are presented as a foundational step toward the future exploration of personalized risk assessment strategies in maternal–fetal medicine.

## Figures and Tables

**Table 1:** Demographic, periodontal, and hematological characteristics of the study population of the ANN and XGBoost models for miscarriage risk prediction.

Parameter	Case Group (*n* = 41) (Mean ± SD)	Control Group (*n* = 41) (Mean ± SD)	*p*-Value	Statistical Significance
Demographic Parameters				
Age (years)	29.42 ± 4.81	28.95 ± 4.53	0.648	NS
Periodontal Parameters				
Clinical Attachment Loss (CAL, mm)	2.84 ± 0.65	1.41 ± 0.38	<0.001 *	Significant (Case High)
Probing Depth (PD, mm)	3.12 ± 0.58	2.16 ± 0.42	<0.001 *	Significant (Case High)
Gingival Index (GI)	1.86 ± 0.44	1.12 ± 0.36	<0.001 *	Significant (Case High)
Plaque Index (PI)	1.79 ± 0.46	1.08 ± 0.32	<0.001 *	Significant (Case High)
Simplified Calculus Index (SCI)	1.42 ± 0.39	0.85 ± 0.27	<0.001 *	Significant (Case High)
Hematological and Inflammatory Parameters				
Plateletcrit (PCT, %)	0.29 ± 0.05	0.24 ± 0.04	0.017 *	Significant (Case High)
Platelet Count (PLT, 10^3^/µL)	284.62 ± 43.15	241.80 ± 39.42	0.012 *	Significant (Case High)
White Blood Cell Count (WBC, 10^3^/µL)	9.28 ± 1.82	10.84 ± 2.15	0.015 *	Significant (Control High)
Neutrophil Count (NC, 10^3^/µL)	6.15 ± 1.38	7.52 ± 1.54	0.008 *	Significant (Control High)
Neutrophil-to-Lymphocyte Ratio (NLR)	2.86 ± 0.68	3.48 ± 0.75	0.028 *	Significant (Control High)
Platelet-to-Lymphocyte Ratio (PLR)	122.45 ± 28.30	118.15 ± 26.40	0.478	NS
Mean Platelet Volume (MPV, fL)	10.24 ± 1.12	9.85 ± 0.94	0.092	NS
Monocyte-to-Lymphocyte Ratio (MLR)	0.24 ± 0.06	0.22 ± 0.05	0.115	NS
Systemic Immune-Inflammation Index (SII)	812.40 ± 215.30	786.10 ± 198.50	0.564	NS

* Values are presented as Mean ± SD. NS: Not Significant (*p* > 0.05). Statistically significant differences (*p* < 0.05) are highlighted.

**Table 2:** Comprehensive statistical performance comparison of the ANN and XGBoost models for miscarriage risk prediction.

Metric	ANN	XGBoost
MSE	2.74 × 10^−2^	2.35 × 10^−2^
RMSE	1.65 × 10^−1^	1.53 × 10^−1^
MAE	7.61 × 10^−2^	1.15 × 10^−1^
R^2^	0.89209	0.92088
D	0.971	0.972
MoD_av_ (%)	2.29	2.36

**Table 3:** Detailed classification performance metrics and ROC analysis results for the developed ANN and XGBoost models in predicting miscarriage risk.

Metric	ANN	XGBoost
AUC (Area Under Curve)	0.9921	1.0000
Sensitivity	1.0000	1.0000
Specificity	0.9750	1.0000
PPV (Pos. Predictive Val.)	0.9762	1.0000
NPV (Neg. Predictive Val.)	1.0000	1.0000
Accuracy	0.9877	1.0000
F1-Score	0.9879	1.0000

## Data Availability

The datasets generated and/or analyzed during the current study are available from the corresponding author upon reasonable request.

## Abbreviations

AI	Artificial Intelligence
ANN	Artificial Neural Network
CAL	Clinical Attachment Loss
CBC	Complete Blood Count
CRP	C-reactive protein
GI	Gingival Index
ICC	Intraclass Correlation Coefficient
LMP	Last Menstrual Period
MLR	Monocyte-to-Lymphocyte Ratio
MPV	Mean Platelet Volume
MPV/PLT	Mean Platelet Volume to Platelet Ratio
NC	Neutrophil Count
NLR	Neutrophil to Lymphocyte Ratio
PCT	Plateletcrit
PD	Probing Depth
PI	Plaque Index
PLR	Platelet to Lymphocyte Ratio
PLT	Platelet Count
SCI	Simplified Calculus Index
SII	Systemic Immune-Inflammation Index
WBC	White Blood Cell
